# Developing Age-Friendly Programs in Greater Manchester, UK: Understanding the Role of Social Infrastructure

**DOI:** 10.1093/geroni/igaa057.2388

**Published:** 2020-12-16

**Authors:** Sophie Yarker, Chris Phillipson

**Affiliations:** 1 University of Manchester, University of Manchester, United Kingdom; 2 Manchester Institute for Collaborative Research into Ageing, Manchester, United Kingdom

## Abstract

This paper considers the critical role of social infrastructure in building age-friendly communities. Drawing on two neighbourhood projects, the paper explores the benefits which different types of social connections bring for older people and the types of spaces in which these connections are produced. It provides support for the importance of ‘natural neighbourhood networks’(Gardner, 2011) by demonstrating how everyday encounters help promote informal networks of support. Following Klinenberg’s (2018) analysis of the importance of social infrastructure, the paper argues that the decline of local high streets, closure of libraries, and cuts to the maintenance of green spaces, reduce opportunities for face-to-face social interactions. The paper presents findings from two studies illustrating the importance of social infrastructure in supporting new forms of community action amongst older people. The paper concludes that that the value of social interactions that occur in everyday mundane spaces needs greater emphasis in public policy.

